# Supplementary figures and images for: A highly conserved host lipase deacylates oxidized phospholipids and ameliorates acute lung injury in mice
Source: eLife. 2021 Nov 16;10:e70938. doi: 10.7554/eLife.70938 (PMC8594946; doi:10.7554/eLife.70938)

Figure\_1\_source\_data\_2

Fig. 1D

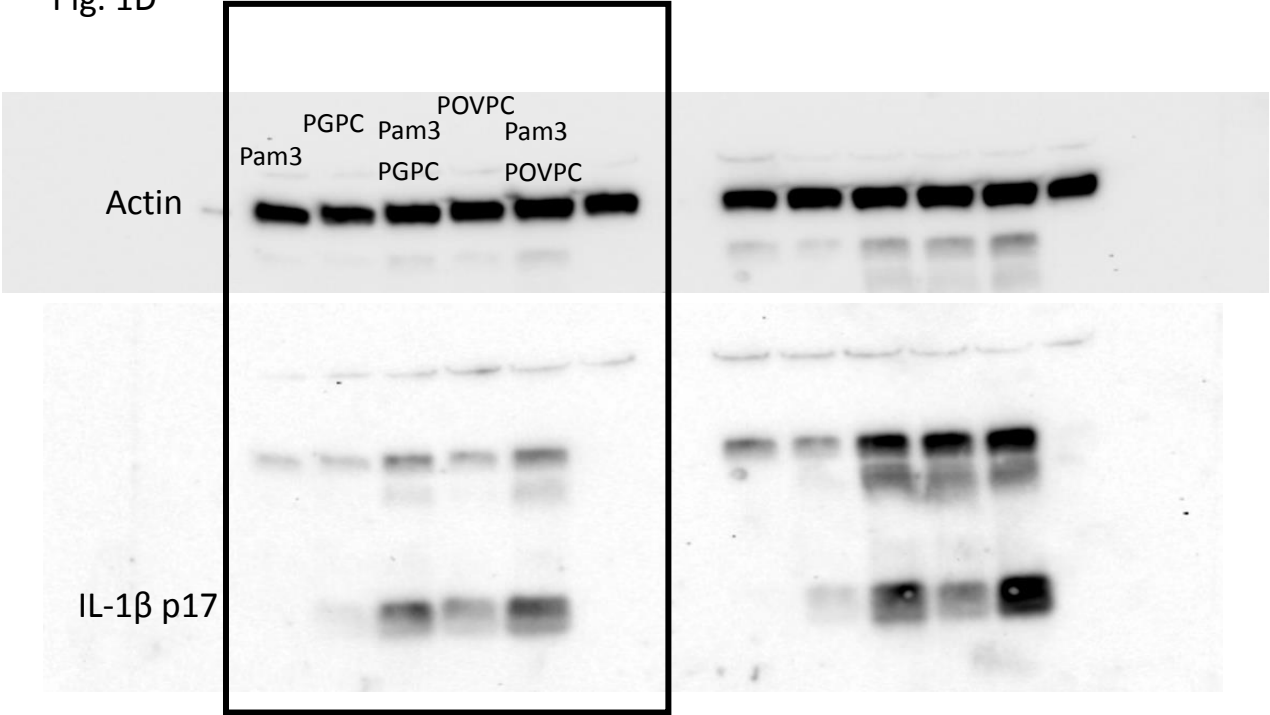

Fig. 1F

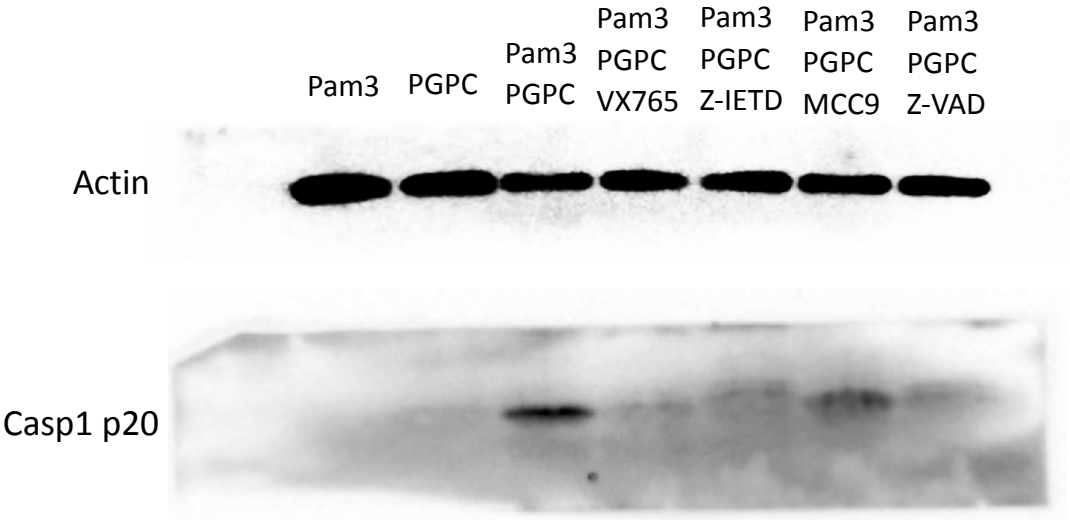

Supplement: Figure 1—source data 2. [file elife-70938-fig1-data2.pdf]

Figure\_1\_figure\_supplement\_1\_source\_data\_2

Fig. 1--figure supplement 1 B

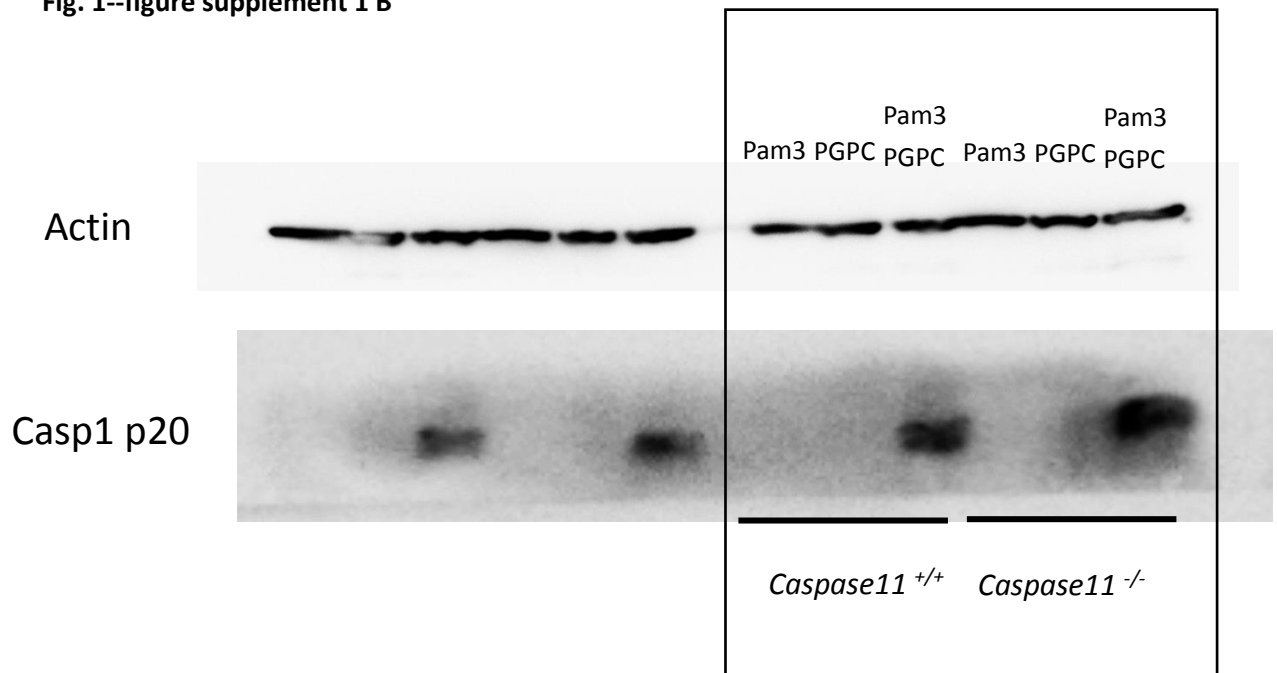

Supplement: Figure 1—figure supplement 1—source data 2. [file elife-70938-fig1-figsupp1-data2.pdf]

Fig. 2F

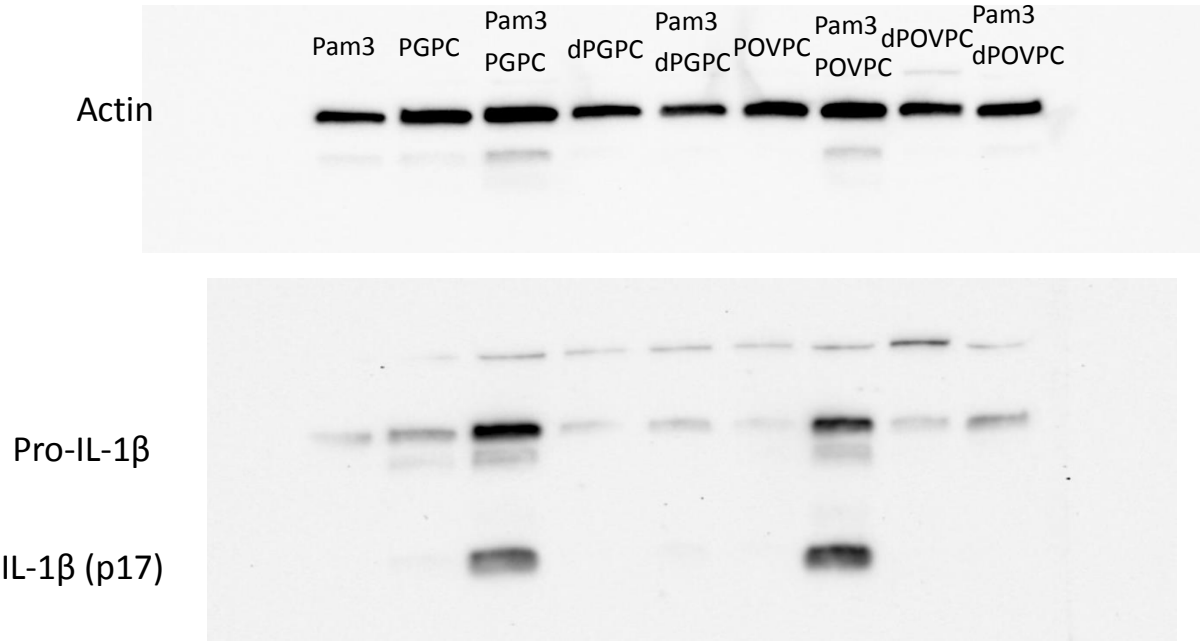

Supplement: Figure 2—source data 2. [file elife-70938-fig2-data2.pdf]

Figure\_3\_source\_data\_2

Fig. 3C

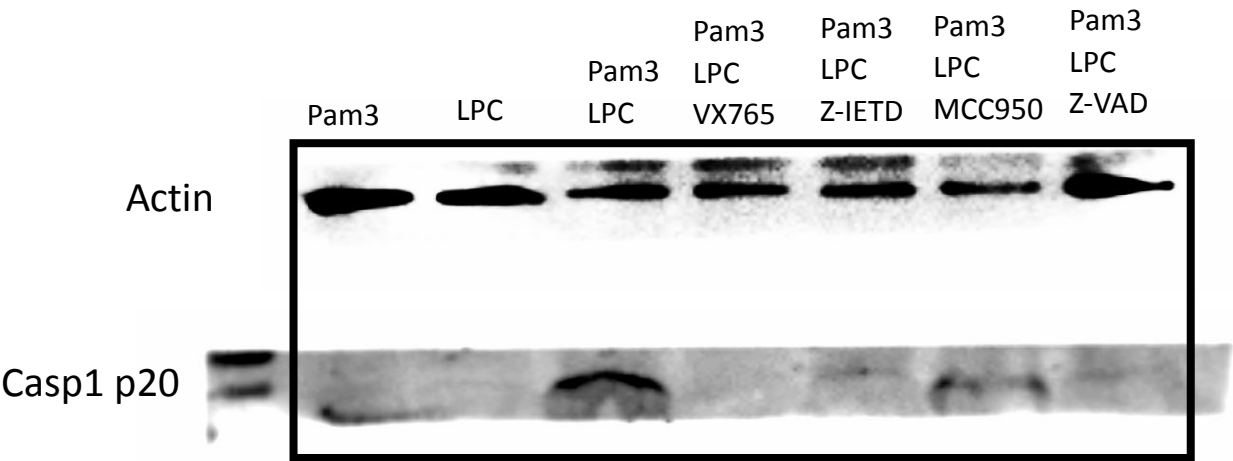

Fig. 3E

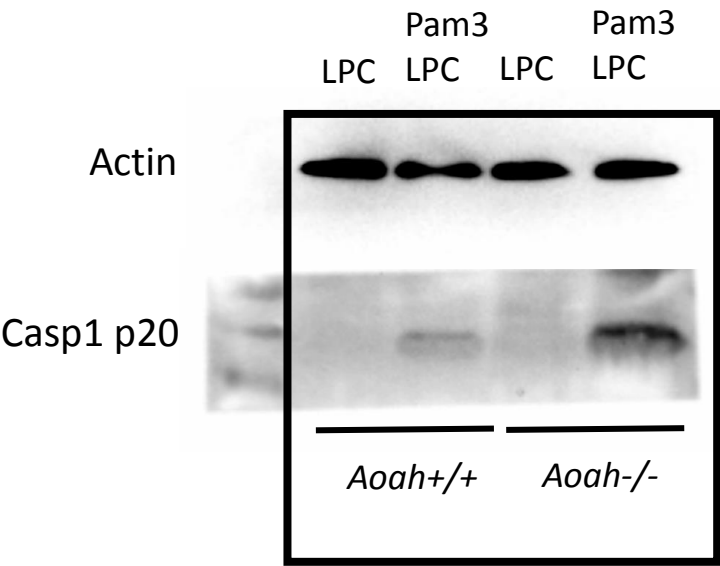

Supplement: Figure 3—source data 2. [file elife-70938-fig3-data2.pdf]

**Fig. 4D**

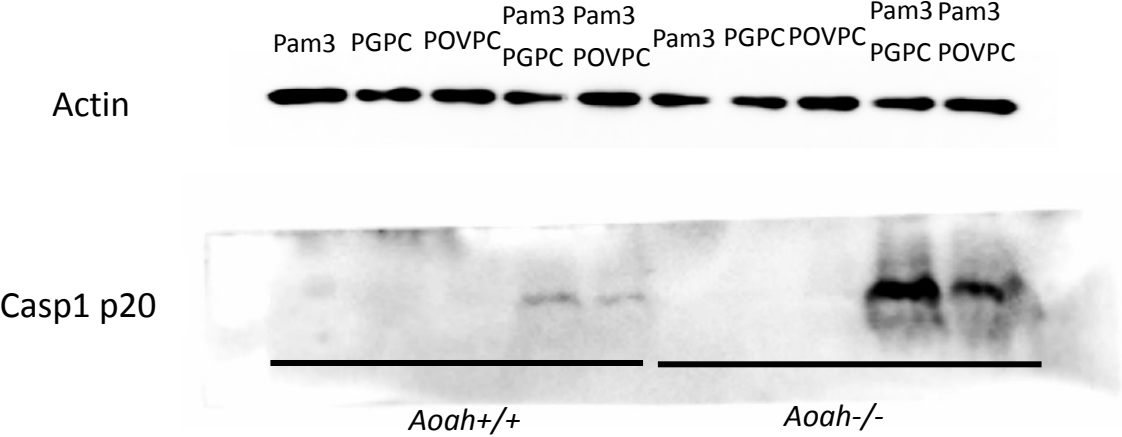

**Fig. 4G**

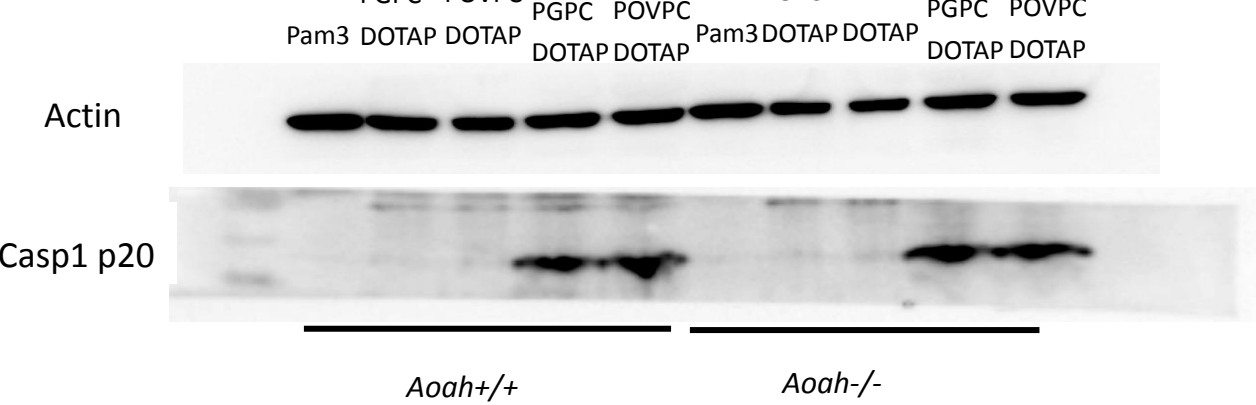

**Fig. 4I**

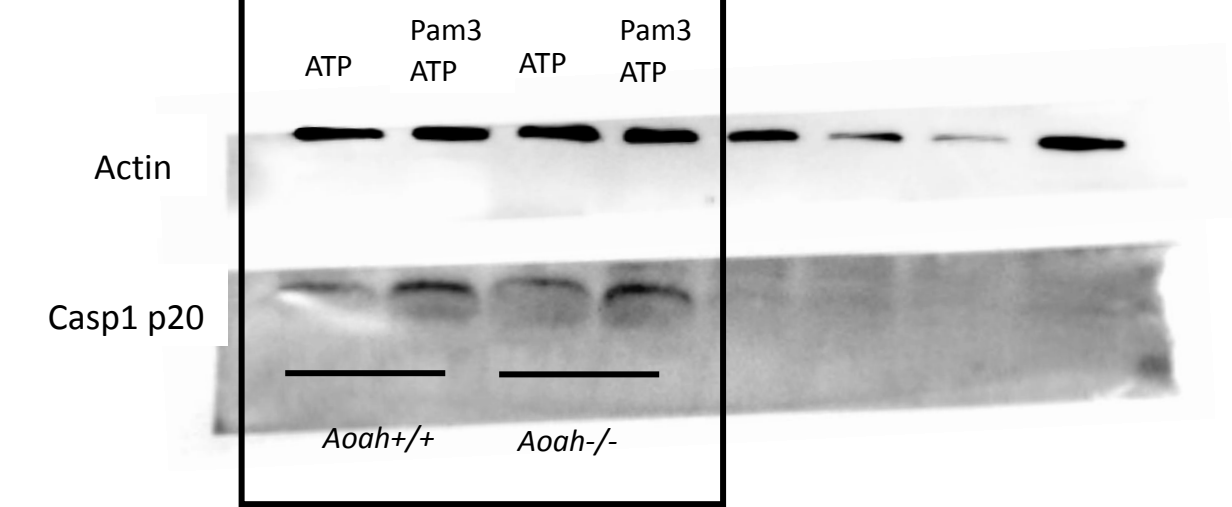

Supplement: Figure 4—source data 2. [file elife-70938-fig4-data2.pdf]
