# Supplementary material for: A highly conserved host lipase deacylates oxidized phospholipids and ameliorates acute lung injury in mice
Source: eLife. 2021 Nov 16;10:e70938. doi: 10.7554/eLife.70938 (PMC8594946; doi:10.7554/eLife.70938)
Supplement: Figure 7—source data 2. [file elife-70938-fig7-data2.pdf]

Figure_7_source_data_2

Fig. 7E

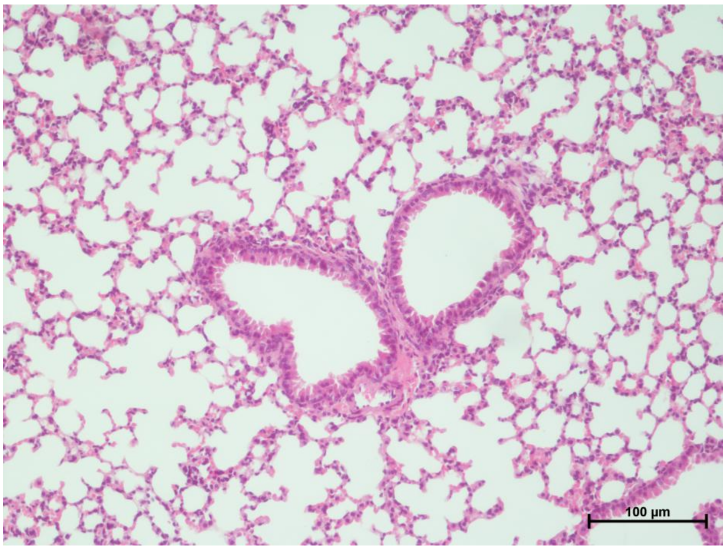

Aoah$^{+/+}$ PBS

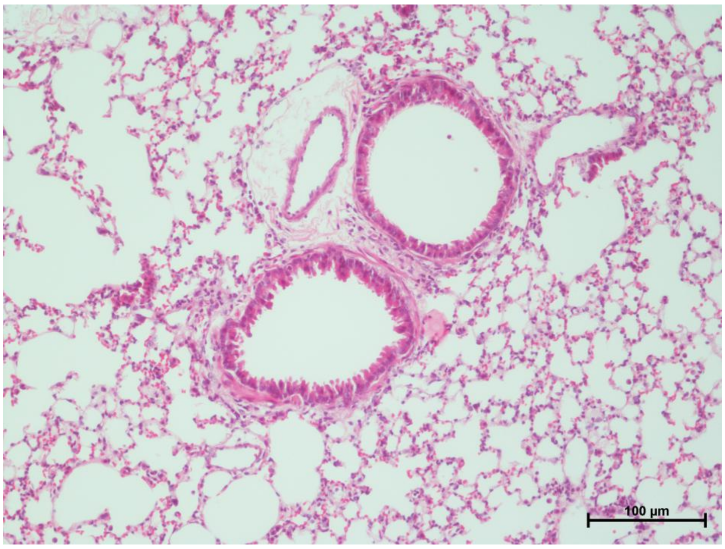

Aoah$^{-/-}$ PBS

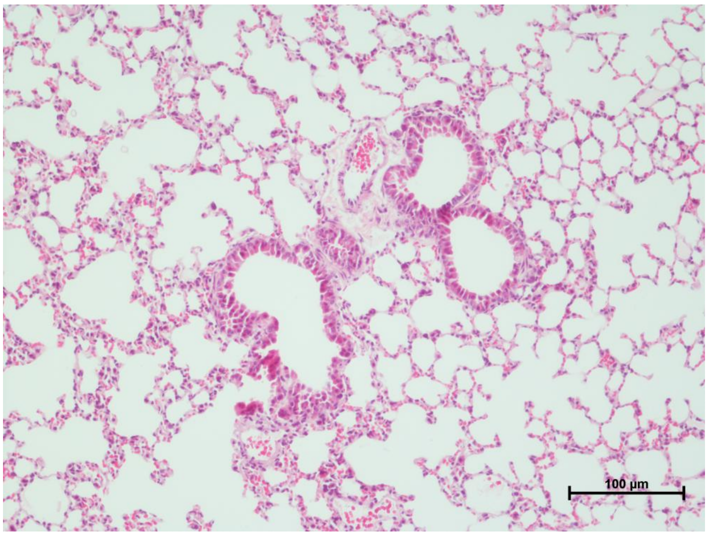

Aoah$^{+/+}$

HCl+Ventilation

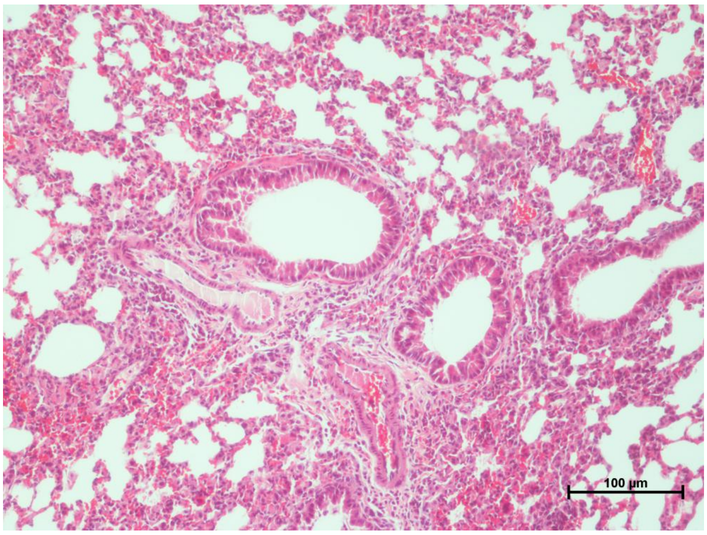

Aoah$^{-/-}$

HCl+Ventilation
